# Feather steroid hormone concentrations in relation to age, sex, and molting time in a long‐distance migratory passerine

**DOI:** 10.1002/ece3.5447

**Published:** 2019-07-23

**Authors:** Marie Adámková, Zuzana Bílková, Oldřich Tomášek, Zdeněk Šimek, Tomáš Albrecht

**Affiliations:** ^1^ Department of Botany and Zoology, Faculty of Science Masaryk University Brno Czech Republic; ^2^ Research Centre for Toxic Compounds in the Environment, Faculty of Science Masaryk University Brno Czech Republic; ^3^ Institute of Vertebrate Biology Czech Academy of Sciences Brno Czech Republic; ^4^ Department of Zoology, Faculty of Science Charles University in Prague Prague Czech Republic

**Keywords:** barn swallow, feather corticosterone, feather testosterone, keratinous matrix, liquid chromatography–tandem mass spectrometry, ptilochronology, stress

## Abstract

In birds, concentrations of testosterone (T) and corticosterone (Cort) are closely connected with many morphological, behavioral, and other physiological traits, including reproduction, metabolism, immunity, and fitness. The direction of the effect of these hormones on above‐mentioned traits, and the potential feedback between hormones are in general unclear; in addition, knowledge on how age and sex can affect T and Cort concentrations is still inconsistent. Our study used a novel method to analyze testosterone and corticosterone in feathers (T_f_, Cort_f_) based on the precolumn chemical derivatization of hormones before liquid chromatography–tandem mass spectrometry (LC‐MS/MS) analysis. Unlike previously used methods (RIA, EIA), our analytical procedure allows simultaneous analysis of both hormones from small amounts of feathers (4–25 mg) and, thus, overcomes the problem of insufficient detection limits. We applied this method to reveal associations between T_f_ and Cort_f_ hormone concentrations and feather growth, age, and sex in feathers grown during the postbreeding (flanks) and prebreeding (tails) periods in barn swallows (*Hirundo rustica*). There was neither a correlation between prebreeding and postbreeding T_f_, nor between prebreeding and postbreeding Cort_f_. Tail Cort_f_ concentrations were negatively associated with tail feather growth rates. Feather hormone concentrations were correlated in the prebreeding period, negatively in males but positively in females. Both Cort_f_ and T_f_ were higher in young birds compared to older ones, indicating either an age‐related decrease in hormone concentrations within individuals, or the selective disappearance of individuals with high steroid concentrations. Males and females did not differ in Cort_f_, but T_f_ concentrations were higher in males than females, particularly during the prebreeding period. In this study, we provide an effective method for analyzing hormones in feathers in an ecological context, especially in situations when the total amount of feathers available for the analysis is limited.

## 1 INTRODUCTION

Steroid hormones are key endocrine mediators of individual fitness that regulate investments in various reproduction and survival‐related traits according to environmental conditions and individual physiological state. In birds, T and Cort are commonly measured steroid hormones in ecological studies. Their concentrations have been shown to reflect individual condition, breeding state, feather growth, affect immune system, reproduction, and control metabolism, and can be dependent on the sex and age of the studied individuals (Ducrest, Keller, & Roulin, 2008; Hau & Goymann, 2015; Kempenaers, Peters, & Foerster, 2008).

A frequent question of many eco‐physiological studies is how T and Cort concentrations change in relation to age and/or sex (Monclús et al., 2017; Wilcoxen, Bridge, Boughton, Hahn, & Schoech, 2013). Previous studies based on analyses of plasma have shown that T concentrations are higher in males compared to females, following its role as the main sexual hormone in males responsible for their breeding behavior and morphological and other physiological traits (reviewed in Kempenaers et al., 2008). Compared with T, sex differences in Cort are less obvious (Fairhurst et al., 2015; Monclús et al., 2017). Large seasonal variations in Cort levels circulating in the blood, the sampling of the analyzed matrix in different periods (Romero, Ramenofsky, & Wingfield, 1997), together with a possible shift in the relationship between Cort and fitness during the breeding season (reviewed in Bonier, Martin, Moore, & Wingfield, 2009) could be the reasons for the absence of a clear sex pattern in Cort concentrations. In addition, both hormones may change their levels depending on the age of individuals, with decreasing levels of both T and Cort reported in most studies (Heidinger, Nisbet, & Ketterson, 2006; Wilcoxen et al., 2013). An increase in hormones during adolescence, following by a decay in later age, could be a result of reproductive senescence (Reed et al., 2008), or an immunocompetence handicap, whereby individuals with high initial steroid concentration levels disappear from the population (Folstad & Karter, 1992).

Testosterone and Cort levels may correlate with each other (reviewed in Roberts, Buchanan, & Evans, 2004). While T stimulates reproduction, Cort inhibits sexual behavior (reviewed in Adkins‐Regan, 2005) leading to the expectation of a negative association between T and Cort concentrations, at least in breeding individuals. Experimental studies, however, indicate that T increases Cort levels and that seasonal increases in both T and Cort are positively correlated (reviewed in Braude, Tang‐Martinez, & Taylor, 1999). Whether similar associations also appear during the nonbreeding season remains unknown.

Most studies to date have used plasma to estimate hormone concentration levels. Hence, our knowledge on sex and age differences in steroid hormone concentrations are often restricted to short‐term patterns in circulating steroid hormone concentrations, as plasma Cort levels increase from basal levels 2–3 min after the initiation of stress stimulus (Romero & Reed, 2005). Feces are another biological material that can be used—in this case, for the analysis of steroid hormone metabolites that reflect hormonal levels over a period of several hours before defecation (Palme, 2005). Interestingly, steroid hormones are also incorporated into skin derivatives such as growing feathers during molting over a period of several days or weeks (Bortolotti, Marchant, Blas, & German, 2008). In birds, feathers could, therefore, provide information about hormonal levels over the entire period of their growth (i.e., up to several weeks; for details about the relationship between feather and plasma concentrations, please see Appendix S1). The capability to analyze hormone profiles from different feather types molting at different times of the year (Boves, Fairhurst, Rushing, & Buehler, 2016) provides an opportunity to assess potential sex or age differences in steroid hormone concentrations in individuals outside the breeding season, and to evaluate the consistency of the endocrine phenotype throughout the year, including relationships between Cort and T or possible carry‐over effects that wintering conditions may have on reproductive success in the subsequent breeding season (Harms et al., 2015).

**Figure 1:**
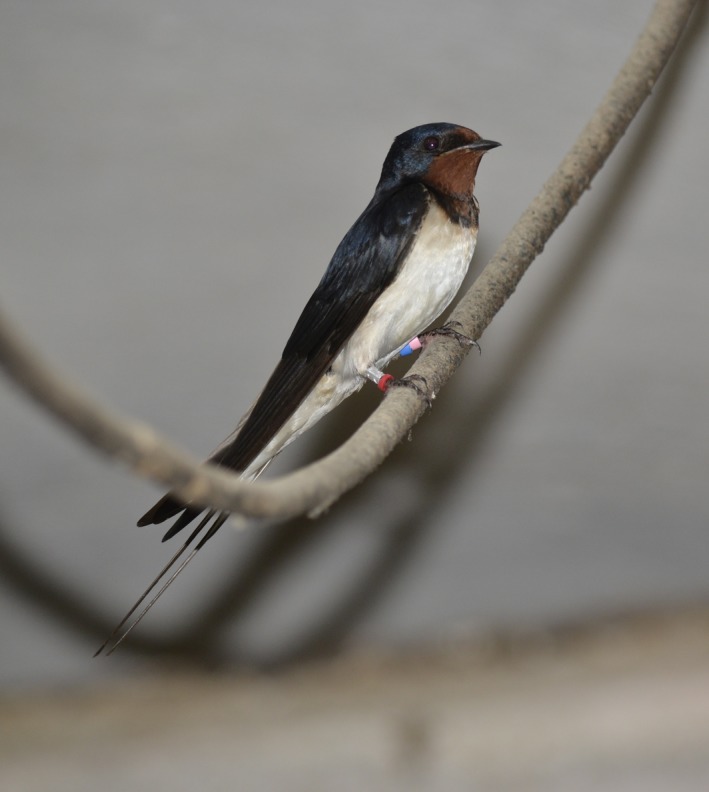
The European barn swallow (*Hirundo rustica rustica*), adult male. Photograph taken by Oldřich Tomášek

Recently, LC‐MS/MS has emerged as a novel method for quantifying steroid hormone concentrations in feathers (Berk, McGettrick, Hansen, & Breuner, 2016; Koren et al., 2012; for details about feather hormones analyses using LC‐MS/MS, please see Appendix S2). We modified the LC‐MS/MS method by performing the precolumn chemical derivatization of feather hormones (for details, please see Bílková, Adámková, Albrecht, & Šimek, 2019). Here, we utilized this method to investigate how hormones deposited in two feather types (T_f_, Cort_f_) molting at different times of the year (flank feathers in postbreeding, tail feathers in prebreeding periods, respectively; Jenni & Winkler, 1994; Rubolini, Massi, & Spina, 2002) are associated with sex, age, and tail feather growth rates in a small migratory passerine, the European barn swallow (*Hirundo rustica rustica*; Figure 1). We tested the following predictions: (a) T_f_ concentrations are in general elevated in males compared to females, particularly during the prebreeding period (following the same pattern as in plasma; Goymann & Wingfield, 2014); (b) feather growth rates are slower in individuals with both elevated Cort_f_ and T_f_ reflecting the negative effect of both hormones on feather growth (Day, McBroom, & Schlinger, 2006; Jenni‐Eiermann, Helfenstein, Vallat, Glauser, & Jenni, 2015); (c) Cort_f_ and T_f_ levels are age‐dependent as consequence of senescence (Reed et al., 2008), or reduced survival of individuals with high hormone concentrations (Folstad & Karter, 1992); and (d) there is an association between Cort_f_ and T_f_ in the prebreeding and postbreeding periods, seeing that we expect negative correlation between these hormones at least in prebreeding concentrations in males, with respect to seasonal dynamics of T (Kempenaers et al., 2008), and negative effect of Cort to reproduction (Adkins‐Regan, 2005).

## 2 MATERIALS AND METHODS

### 2.1 Experimental protocol

Feather samples for the analysis of feather hormone concentrations from flanks (range 17–25 mg, mean 24.7 mg) and inner left tail feathers (range 4–7 mg, mean 5 mg) were collected by gently plucking feathers from 115 barn swallow individuals during the breeding period (from April to July) in 2014 (for details about the field study, please see Appendix S3). A typical sample of flanks weighing about 25 mg contained 42–46 feathers (*n* = 5).

Feather growth rates (hereinafter FGR) were estimated on the basis of the growth bar analysis of tail feathers available for the entire dataset (see above). Each sampled tail feather was weighed, and the length of both the whole feather and the rachis were measured to the nearest 0.01 mm by a digital calliper. A segment on the distal part of the feather containing about 10 pairs of light and dark bars was measured. FGR was calculated as the ratio of the segment length in millimeters to the number of pale/dark bar pairs in the segment (Saino et al., 2012), thus providing the feather length (in mm) grown per one day. The repeatability, estimated by the analysis of 13 feathers measured twice, was 0.96 (*F*_12,13_ = 50.7, *p* < 0.001).

### 2.2 Extraction of hormones and extract processing

The calamus was removed from each tail feather and the feather was weighed and minced into pieces of <1 cm^2^ with scissors. Flank feathers were only weighed as they were always <1 cm^2^ in size and softer. Samples were then pulverized in a ball mill (Mixer Mill MM 200; Retsch) at 30 Hz for 120 min using 3 mm stainless steel grinding balls (22.455.0002; Retsch). Subsequently, 1 ml of HPLC‐grade methanol (494291; Sigma‐Aldrich) was added to the pulverized samples. Samples were then shaken using an orbital shaker in a horizontal position at 450 rpm for 24 hr. After shaking, samples were centrifuged at 2,500 *g* for 5 min and supernatants were collected using a pipette. Pellets were resuspended in 1 ml of methanol, the sample was centrifuged again, and both extracts were mixed together. The extracts were spiked with 10 μl of a mixture of internal standards at a concentration of 600 μg/ml for each deuterated hormone (D‐5822/0,005 n.v corticosterone‐2,2,4,6,6,17α,21,21‐d8, D‐5917/0,01 n.v testosterone‐16,16,17‐d3; C/D/N Isotops Inc.). The methanol was evaporated under a stream of nitrogen and the remainder was dissolved in 2 ml of a mixture of methanol and water (5:95). The dissolved extract was then transferred to SPE columns (Bond Elut C18 SPE cartridges, 3 ml, 100 mg sorbent, end‐capped; Agilent Technologies) preconditioned by methanol and a mixture of methanol and water (5:95). The SPE column was then washed with 2 ml of deionized water. The elution of target analytes was realized by the addition of 2 ml of methanol to vials. The eluates from SPE columns were almost evaporated to dryness under a stream of nitrogen and the residues of eluates were consequently transferred into micro‐vials and dried completely under a stream of nitrogen.

### 2.3 The derivatization and quantification of hormones

For hormone derivatization, 50 µl of derivatization reagent working solution QAO Reagent (Amplifex™ Keto reagent; AB SCIEX) was added into each micro‐vial (for details about the derivatization technique, please see Star‐Weinstock, Williamson, Dey, Pillai, & Purkayastha, 2012). After 120 min of heating at 65°C, 10 µl of deionized water was added before vortex mixing. Target analytes were analyzed by LC‐MS/MS with electrospray ionization. An Agilent 1200 chromatographic system (Agilent) equipped with a vacuum degasser, binary pump, autosampler, and column thermostat was connected online to an ESI/QqQ Agilent Triple Quad 6410 mass spectrometer (Agilent). An ACE 3 C18 analytical column (150 mm × 2.1 mm i.d., 3 µm) with an ACE 3 C18 integrated guard column (2.1 mm × 10 mm, 3 μm; ACE, Scotland, UK) were used for analytical separation. The column temperature was set to 25°C. The chromatographic/mass spectrometric system was controlled by Mass Hunter software. For further details of LC‐MS/MS analyses, see Appendix S4 and Table S1. T_f_ and Cort_f_ were expressed as pg of hormones per 1 g of feathers in both feather types, seeing strong correlations between concentrations expressed per gram and per weight in tail feathers (*n* = 32, *r* = 0.95 for Cort, *r* = 0.93 for T; M. Adámková, unpublished data).

### 2.4 Statistical analyses

All data processing and statistical analyses were performed in R 3.2.3 software (R Core Team, 2016). All repeatabilities were calculated from one‐way ANOVA with the individual as a factor (Lessells & Boag, 1987). Hormone concentration data were log transformed in order to achieve a normal distribution (also see Fairhurst et al., 2015). To evaluate the consistency of the feather hormonal profile in the postbreeding and prebreeding periods, we estimated the within‐individual repeatability of pre‐ and postbreeding hormone concentrations. Relationships between Cort_f_ and T_f_ were tested separately for both sexes using Pearson product moment correlation coefficients on log‐transformed hormone concentrations.

To evaluate the relationships between hormone concentrations and their biologically relevant predictors, we used linear models with hormone concentrations (log‐transformed) as dependent variables and sex, age, and their two‐way interaction as explanatory variables. Because hormone detectability may increase with sample mass (Berk et al., 2016), sample weight was included in all initial models as a covariate. Linear modeling was also used to analyze the association between FGR as a dependent variable and Cort_f_, T_f_, age, sex, and their two‐way interactions as explanatory variables. Age was included in the model as a continuous variable. Because there were only five 4‐year‐old individuals, four 5‐year‐old individuals and one 6‐year‐old individual in our dataset, data from these birds were lumped together, hence age ranged between one and four in our analysis. Because the association between age and hormone concentration could be nonlinear, we also evaluated their second‐order polynomial effects in addition to the linear ones. Initial full models were simplified by removing nonsignificant predictors, starting with interaction terms, to obtain a minimal adequate model (hereinafter MAM; Crawley, 2013). For the purpose of data presentation, we centered all dependent variables in the models containing significant interaction terms in order to enable the main effects to be properly interpreted without the need to remove interaction terms from the models.

## 3 RESULTS

### 3.1 LC‐MS/MS analysis

**Figure 2:**
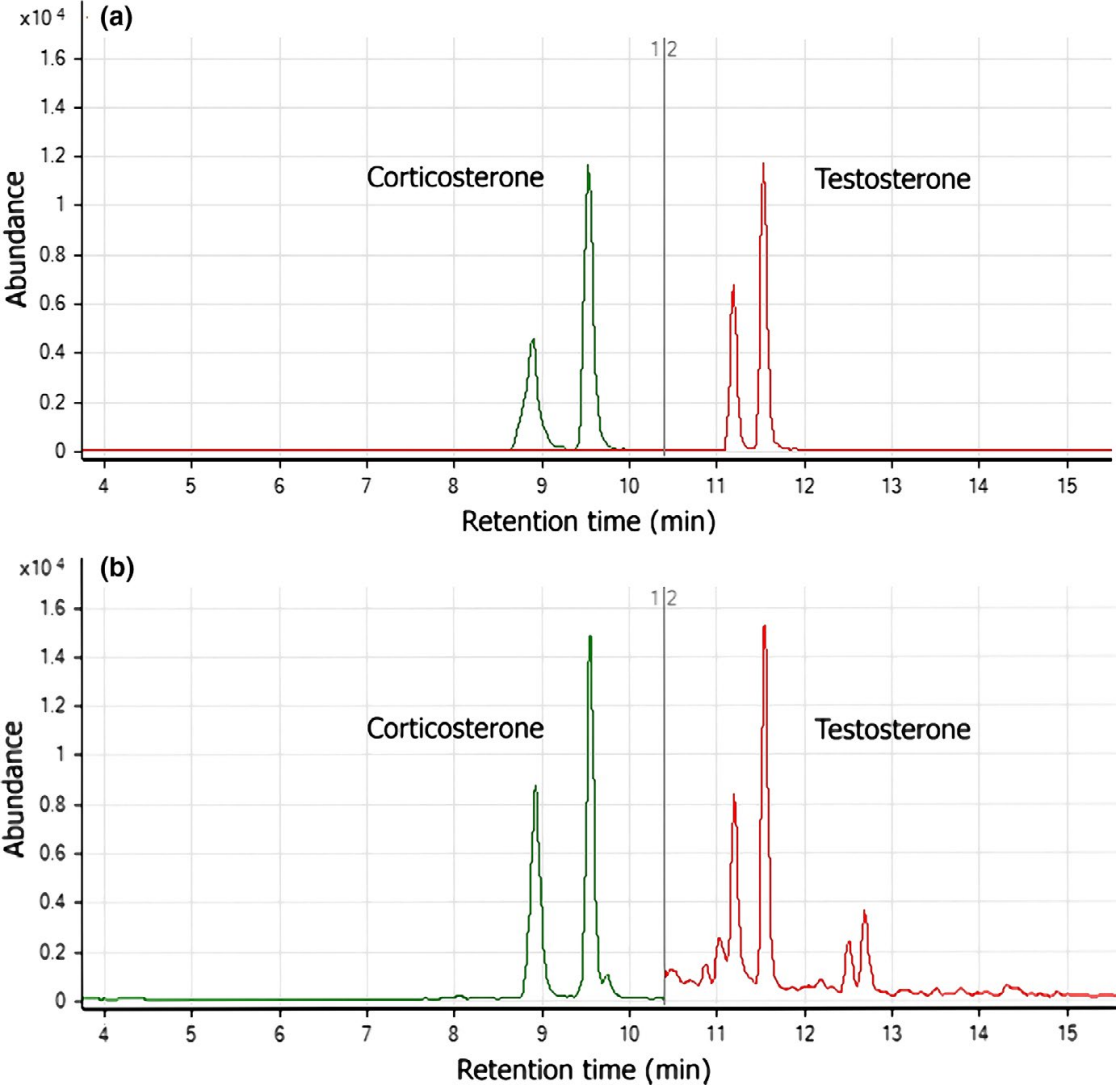
Chromatograms of pairs of isomers of testosterone and corticosterone (a) standard solution and (b) extract from feathers

We were unable to identify any chromatographic peaks corresponding to T and Cort in the chromatograms of extracts from the model set of real feather samples obtained during the preliminary validation of the LC‐MS/MS method without precolumn derivatization. Therefore, in our study, we exclusively used the method with precolumn derivatization, resulting in the elution of two pairs of chromatographic peaks (for T and for Cort, see Figure 2). Linear calibration curves were obtained in the range of 250–2500 pg/ml (for validation parameters of analyses of T and Cort, please see Table S2). LOQs of analytes were 0.83 pg/injection for Cort and 0.25 pg/injection for T. Using this method, we were able to detect and quantify Cort_f_ and T_f_ concentrations in all 115 samples (ranging in weight between 4 and 25 mg).

The repeatability of the whole process of Cort_f_ and T_f_ analysis, including the extraction of hormones from the matrix, was estimated by means of the analysis of flank feathers from 10 individuals. The feather sample from each individual was divided into two subsamples of the same weight (25 mg) and analyzed separately. The repeatability was 0.79 for Cort_f_ (*F*_9,10_ = 8.328, *p* = 0.001) and 0.91 for T_f_ (*F*_9,10_ = 21.42, *p* < 0.001). It was not possible to perform a similar analysis for tail feathers as the amount of material available was limited.

### 3.2 Hormone levels repeatability: correlation between Cort_f_ and T_f_ concentrations

The concentration ranges of Cort_f_ and T_f_ in both feather types are provided in the Table S3. Hormone levels from postbreeding (flanks) and prebreeding (tails) periods were not repeatable within individual birds (*R* = 0.57, *F*_113,114_ = 1.34, *p* = 0.06 for Cort_f_; *R* = 0.36, *F*_113,114_ = 0.57, *p* = 0.999 for T_f_). There was no correlation between Cort_f_ and T_f_ concentrations in the postbreeding period in either sex (males: *r* = −0.15, *p* = 0.21; females: *r* = −0.08, *p* = 0.62). In contrast, prebreeding Cort_f_ and T_f_ were positively correlated in females and negatively in males (males: *r* = −0.34, *p* = 0.004; females: *r* = 0.43, *p* = 0.003).

### 3.3 Age, sex, and Cort_f_

**Figure 3:**
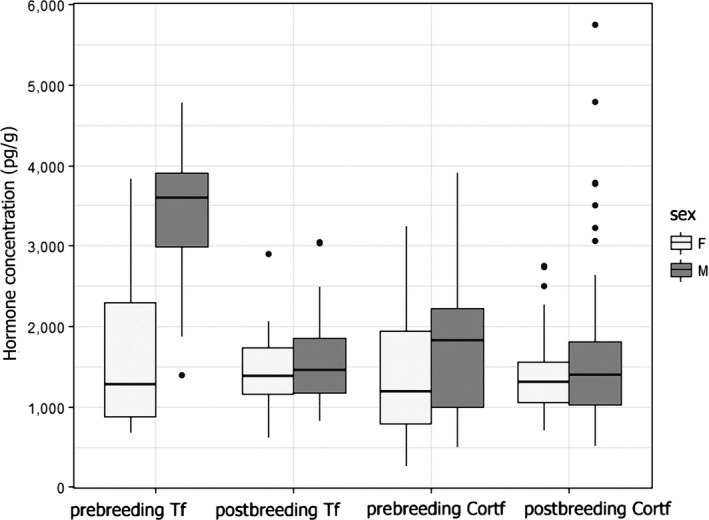
Postbreeding and prebreeding concentrations of feather hormones in both females and males

In the next step, we analyzed variations in postbreeding and prebreeding feather hormones in relation to age and sex, with sample mass included in the models as a covariate. The *age × sex* interaction was not important in explaining the variation of postbreeding Cort_f_ concentrations (comparison of models with and without the interaction term: *F* = 0.22, ΔDf = 1, *p* = 0.64). Similarly, sample mass was not associated with postbreeding Cort_f_ and there were no sex differences in postbreeding Cort_f_ (full model in Table S4). The MAM contained only age (*F*_1,113_ = 4.5, *p* = 0.036, slope = −0.085 ± 0.04 [*SE*]) and explained 4% of variation. Tail Cort_f_ concentrations showed a similar pattern: the *age* × *sex* interaction term was not important (*F* = 0.47, ΔDf = 1, *p* = 0.5). The full model is provided in the Table S4. The MAM contained only age (*F*_1,113_ = 16.93, *p* < 0.001, slope = −0.211 ± 0.051 [*SE*]) and explained 13% of variation. There was no difference between sexes in prebreeding and postbreeding Cort_f_ (Figure 3).

In the analyses of both pre‐ and postbreeding Cort_f_ concentrations, we also evaluated the possibility that the association between age and Cort_f_ is actually nonlinear, by replacing the linear effect of age in the MAM by its second‐order polynomial. The analysis indicated linear associations between age and Cort_f_ (comparison of linear and polynomial models in the postbreeding period: *F* = 0.19, ΔDf = 1, *p* = 0.666; prebreeding period: *F* = 1.82, ΔDf = 1, *p* = 0.18).

### 3.4 Age, sex, and T_f_

Postbreeding T_f_ was associated differently with age in males and females (comparison of models with and without the *age × sex* interaction term: *F* = 8.16, ΔDf = 1, *p* = 0.005). See Table S4 for results of the full model. Sample mass was not associated with variation in postbreeding T_f_ (*F* = 0.39, ΔDf = 1, *p* = 0.534). The MAM involved age, sex, and the *age × sex* interaction term and explained 9% of variation (presented in Table 1). Separate analyses for each sex indicated that postbreeding T_f_ was not associated with age in females (*F*_1,43_ = 2.68, *p* = 0.109, slope = 0.071 ± 0.043 [*SE*]); however, there was a negative correlation in males (*F*_1,67_ = 6.46, *p* = 0.013, slope = −0.11 ± 0.043 [*SE*]). There was no evidence for a polynomial relationship either in females (*F* = 1.12, ΔDf = 1, *p* = 0.296) or in males (*F* = 3.2, ΔDf = 1, *p* = 0.078). There was no sex difference in postbreeding T_f_ (Figure 3).

**Table 1:** Minimal adequate model of postbreeding T_f_ (log transformed) in relation with sex (female as a reference), age, and *sex × age* interaction

	Estimate	*SE*	*df*	*F*	*p*
(Intercept)	7.21	0.048			
Sex	0.069	0.061	1	0.775	0.35
Age (centered)	0.068	0.044	1	0.491	0.485
Sex:age (centered)	−0.177	0.062	1	8.156	0.005

The *age × sex* interaction was unimportant in explaining prebreeding T_f_ (*F* = 0.48, ΔDf = 1, *p* = 0.49). See Table S4 for results of the full model. Sample mass was not associated with variation in prebreeding T_f_ (*F* = 1.43, ΔDf = 1, *p* = 0.235). Similarly, age was not associated with T_f_, and further analysis indicated that there was also no polynomial age effect on prebreeding T_f_ (addition of the second‐order polynomial instead of linear age effect did not improve the model: *F* = 0.002, ΔDf = 1, *p* = 0.964). The MAM contained only sex (*F*_1,113_ = 155.98, *p* < 0.001, slope = 0.892 ± 0.071 [*SE*]) and explained 58% of variation. There was a clear sex difference in prebreeding T_f_, with males having much higher concentrations than females (Figure 3).

### 3.5 Feather hormones and feather growth rate

**Figure 4:**
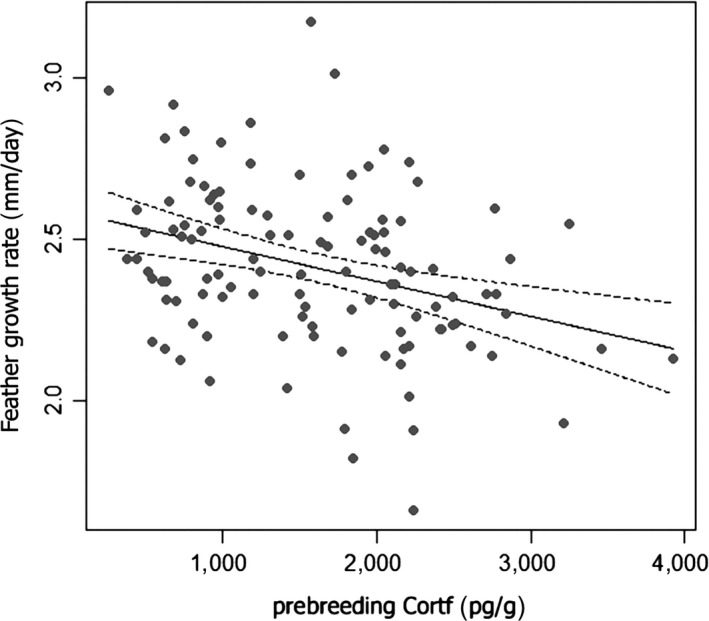
Correlation between feather growth rate and prebreeding Cort_f_. Dashed lines denote confidence intervals around the predicted values, full line is the regression line

We also evaluated the idea that hormone concentrations are correlated with FGR. The full model (see Table S5) evaluating the effects of sex, age, prebreeding Cort_f_ and T_f_, and the interactions between hormone concentrations and sex and hormone concentrations and age, respectively, indicated sex differences in tail FGR and the clear effect of Cort_f_ concentrations on tail FGR. FGR was faster in females than males (2.51 ± 0.23 [*SE*] mm/day in females vs. 2.35 ± 0.24 [*SE*] mm/day in males) and faster in individuals with lower tail Cort_f_. The MAM (presented in Table 2) involved only sex and Cort_f_, not T_f_, and explained 16% of variation in FGR (also see Figure 4).

**Table 2:** Minimal adequate model of feather growth rate in relation to sex (female as a reference) and prebreeding Cort_f_ (log transformed)

	Estimate	*SE*	*df*	*F*	*p*
(Intercept)	3.31	0.286			
Sex	−0.132	0.046	1	8.22	0.005
Prebreeding log(Cort_f_)	−0.113	0.04	1	7.9	0.006

## 4 DISCUSSION

In this study, we analyzed hormone concentrations from feathers grew during the prebreeding (tail feathers) as well as postbreeding (flank feathers) periods, allowing us to track seasonal changes in hormone concentrations over the entire wintering period of individual birds. We selected tail and flank feathers from 115 barn swallow individuals and estimated Cort_f_ and T_f_ concentrations in all samples using LC‐MS/MS with precolumn chemical derivatization. This is in striking contrast to a single previous study that attempted to estimate Cort_f_ and T_f_ in an ecological context using the LC‐MS/MS approach (Koren et al., 2012), where only 16 of 61 samples provided Cort_f_ and 34 out of 35 samples provided T_f_ estimates, even though a much higher feather sample mass was used (21–84 mg). For details about the effect of derivatization on hormone quantification, please see Appendix S5.

To the best of our knowledge, our study is the first attempt to analyze the associations between T and Cort deposited in feathers during the postbreeding and prebreeding periods. Studies based on plasma hormone concentrations indicate that there could be a different association between Cort and T levels (reviewed in Roberts et al., 2004). We found that the negative association between Cort_f_ and T_f_ is, in general, weak (*r* = −0.34) and only appears during the prebreeding period, when males have elevated levels of T (see below). In females, the association was of a similar magnitude, but it was opposite (*r* = 0.43). Despite intense research focusing on the relationship between Cort and T, it is not entirely apparent how these are related. There is some evidence for both a positive (Duffy, Bentley, Drazen, & Ball, 2000; Evans, Goldsmith, & Norris, 2000) and a negative (Duckworth, Mendonca, & Hill, 2001; Quillfeldt, Masello, Strange, & Buchanan, 2006) correlation between plasma Cort and T (but see Hau, Ricklefs, Wikelski, Lee, & Brawn, 2010). Some discrepancy could be due to the possibility that the direction of the association between Cort and T is sex dependent. In males, T is known as the main sexual hormone that is positively correlated with individual quality (Kempenaers et al., 2008) and possibly low Cort levels, whereas increased T levels could be harmful to females (Rutkowska, Cichoń, Puerta, & Gil, 2005) resulting in high Cort levels.

Feather hormone concentrations analyzed from tail (prebreeding) and flanks (postbreeding) were not correlated in our study, indicating inconsistency in hormonal profiles in different periods of the year. Such inconsistency seems to be typical for species in which feathers from different body regions molt at different times of the year (Boves et al., 2016); in other species in which feathers from different body regions molt at the same time, these profiles are consistent (Lendvai, Giraudeau, Németh, Bakó, & McGraw, 2013). According to our expectation, we detected higher levels of T_f_ in both feather types from males when compared with females. This finding is consistent with the function of testosterone as a male sexual hormone (Kempenaers et al., 2008). Concentrations of T_f_ were elevated in feathers molted during the prebreeding period in males only, and this is most probably associated with an increase in T production in the spring, prior to the breeding period, when sexual ornamentation (elongated tail streamers in barn swallows) also develops (Goymann & Wingfield, 2014). The analysis of hormones from feathers molting in various periods of the year may provide interesting information about the dynamics of hormone levels during the annual cycle (Boves et al., 2016), especially in long‐distance migratory birds.

In contrast to T_f_, Cort_f_ concentrations did not differ between males and females. This corresponds well with a previous study that used RIA to estimate Cort_f_ levels in tail feathers in barn swallows (Fairhurst et al., 2015) but not with data available for some other avian species (Fairhurst, Dawson, van Oort, & Bortolotti, 2014). This could be because of changes in Cort levels in dependence on seasonal physiological changes (Romero et al., 1997) that pose different demands on each sex.

There was a decrease in Cort_f_ with age in both sexes, both in the pre‐ and postbreeding period, and there was a decrease in postbreeding T_f_ with age in males only. Hormone levels typically change with age, but the results are inconsistent. For example, some studies of comparatively long‐lived species, such as wandering albatrosses, common terns, and snow petrels, report an increase in plasma basal Cort with age (Angelier, Shaffer, Weimerskirch, & Chastel, 2006), a decrease with age (Heidinger et al., 2006), or no effect with age (Goutte, Antoine, Weimerskirch, & Chastel, 2010). In the latter case, however, there was evidence of differences in stress Cort levels between a group of young and senescent individuals with higher Cort and a group of middle‐aged individuals with lower Cort (Goutte et al., 2010). Basal plasma Cort may not vary with age in short‐lived species (Lendvai, Giraudeau, Bókony, Angelier, & Chastel, 2015). As in stress plasma (see above), Cort_f_ decreased with age in both long‐ and short‐lived species in some studies (Boves et al., 2016; López‐Jiménez et al., 2016), while other studies found Cort_f_ to be independent of age (Grunst, Grunst, Parker, Romero, & Rotenberry, 2014; Strong, Pereira, Shore, Henrys, & Pottinger, 2015). Similarly, there is inconsistency in the direction of the correlation between age and T concentrations (for positive, see e.g., Bautista et al., 2013; for negative, see e.g., Wilcoxen et al., 2013), although most studies found the plasma T concentration to be a trait independent of age, especially in males (reviewed in Kempenaers et al., 2008). To our knowledge, no study has investigated the effect of age on T_f_ concentration.

Elevated levels of T_f_ and Cort_f_ in young birds could be explained either in terms of reproductive senescence (Reed et al., 2008), or by the reduced survival of individuals with high Cort and T levels (Buchanan, 2000; Folstad & Karter, 1992; Koren et al., 2012). Unfortunately, we do not have longitudinal data to evaluate these two scenarios. Barn swallows are relatively short‐lived birds but there is some evidence of reproductive senescence in this species (Møller et al., 2009). We did not find evidence of nonlinear changes in steroid hormone levels with age, but again, we were limited by the lack of individually based longitudinal data.

To test the effect of Cort_f_ on feather growth, we tested whether FGR corresponded to the prebreeding Cort_f_ level analyzed from the same feather. Similarly as in a previous study (Saino et al., 2012), FGR was found to be faster in females than in males. The negative relationships between Cort_f_ and FGR found in our study were consistent with the findings of several previous studies (Jenni‐Eiermann et al., 2015; Romero, Strochlic, & Wingfield, 2005; but see Fairhurst et al., 2014) and were in agreement with the idea that elevated Cort decreases feather quality (Lattin, Reed, DesRochers, & Romero, 2011). Thus, faster FGR and lower Cort_f_ could be signals of less stressful environmental conditions or better stress resistance. Contrariwise, no effect on feather growth rate was found for T_f_ in this study, although a negative effect of high T on feather growth has been documented elsewhere (Day et al., 2006; De Ridder, Pinxten, Mees, & Eens, 2002). The lack of an association between T_f_ and tail feather growth in barn swallows is interesting, as tail streamers seem to function as sexually selected ornaments in this species, and T has a stimulating effect on the development of traits important to mate choice (Saino & Møller, 1994).

## 5 CONCLUSIONS

In this study, using a modified LC‐MS/MS method for the simultaneous quantification of hormones based on chemical derivatization, we examined the effect of both age and sex on T_f_ and Cort_f_ concentrations, the effect of feather hormone concentrations on feather growth, and the relationship between Cort and T. The procedure described here provides for the analysis of relatively small amounts of feathers—for example, a single tail feather of small passerines. Our data show that by using this method, it is possible to evaluate relationships between concentrations of Cort and T over different periods of the individual annual cycle, namely before the breeding season and in postbreeding birds. The estimation of hormone from feathers molting in different periods of the year and their associations with ornament expression on the one hand, and breeding plasma hormone concentrations on the other, will allow for a better understanding of the physiological mechanisms ensuring signal honesty of individual quality via secondary sexual traits as well as allow for a better understanding of potential dynamic feedback (Safran, Adelman, McGraw, & Hau 2008) between individual physiology and ornament expression.

## CONFLICT OF INTEREST

None declared.

## AUTHORS’ CONTRIBUTIONS

TA, MA and OT designed the study; ZŠ, ZB and MA designed the analytical methodology; MA, OT and TA collected samples; MA and ZB analyzed the data; MA and TA performed statistical analysis and led the writing of the manuscript. All authors contributed critically to the drafts and gave final approval for its publication.

## Data Availability

The data supporting the results are archived in the Dryad repository. The doi for our data is https://doi.org/10.5061/dryad.v4bf803.
