# Supplementary material for: Feather steroid hormone concentrations in relation to age, sex, and molting time in a long‐distance migratory passerine
Source: Ecol Evol. 2019 Jul 23;9(16):9018–26. doi: 10.1002/ece3.5447 (PMC6706234; doi:10.1002/ece3.5447)
Supplement: Supplementary file 1 [file ECE3-9-9018-s001.docx]

**Supporting Information**

**Feather steroid hormone concentrations in relation to age, sex, and moulting time in a long distance migratory passerine**

Marie Adámková^(1,2)^, Zuzana Bílková^(2)^, Oldřich Tomášek^(3,4)^, Zdeněk Šimek^(2)^ & Tomáš Albrecht^(3,4)^

^(1)^Department of Botany and Zoology, Faculty of Science, Masaryk University, Brno, Czech Republic; ^(2)^Research Centre for Toxic Compounds in the Environment, Faculty of Science, Masaryk University, Brno, Czech Republic; ^(3)^Institute of Vertebrate Biology, Czech Academy of Sciences, Brno, Czech Republic; ^(4)^Department of Zoology, Faculty of Science, Charles University in Prague, Prague, Czech Republic

**Appendix S1:** Relationship between feather and plasma Cort concentrations

It has been reported, at least in the case of Cort, that feather hormone levels are positively correlated with stress plasma hormone levels, but not with baseline stress plasma hormone levels (Bortolotti, Marchant, Blas, & German 2008). Baseline stress plasma and feather Cort levels do not always need to be correlated, because mild and short elevations in plasma might not become evident in feathers (Fairhurst, Marchant, Soos, Machin, & Clark 2013), although they have similar seasonal dynamics across the breeding season (Bortolotti, Marchant, Blas, & German 2008). Contrariwise, long-term and strong stress events always affect feather Cort, as was shown by manipulative experiments involving the implanting of Cort-releasing pellets during feather growth (e.g. Jenni-Eiermann, Helfenstein, Vallat, Glauser, & Jenni 2015). The analysis of feather hormones thus represents a great advancement in our ability to study the links between stress, sex hormone levels, and feather growth (e.g. Jenni-Eiermann, Helfenstein, Vallat, Glauser, & Jenni 2015) or plumage ornamentation (Fairhurst, Dawson, van Oort, & Bortolotti 2014).

References:

Bortolotti, G. R., Marchant, T. A., Blas, J. & German, T. (2008). Corticosterone in feathers is a long-term, integrated measure of avian stress physiology. *Functional Ecology*, 22, pp.494-500. doi: 10.1111/j.1365-2435.2008.01387.x

Fairhurst, G., Marchant, T., Soos, C., Machin, K. & Clark, R. (2013). Experimental relationships between levels of corticosterone in plasma and feathers in a free-living bird. *Journal of Experimental Biology*, 216(21), pp.4071-4081. doi: 10.1242/jeb.091280

Fairhurst, G., Dawson, R., van Oort, H. & Bortolotti, G. (2014). Synchronizing feather-based measures of corticosterone and carotenoid-dependent signals: what relationships do we expect? *Oecologia*, 174(3), pp.689-698. doi: 10.1007/s00442-013-2830-5

Jenni-Eiermann, S., Helfenstein, F., Vallat, A., Glauser, G. & Jenni, L. (2015). Corticosterone: effects on feather quality and deposition into feathers. *Methods in Ecology and Evolution*, 6(2), pp.237-246. doi: 10.1111/2041-210X.12314

**Appendix S2:** Feather hormones analyses using LC-MS/MS

Compared with the traditionally used radioimmunoassay (e.g. Bortolotti, Marchant, Blas, & German 2008) and enzyme immunoassay (e.g. Jenni-Eiermann, Helfenstein, Vallat, Glauser, & Jenni 2015), LC-MS/MS provides high detection specificity and is not limited to a single analyte per one analysis. In the only study published so far, this approach enabled the simultaneous detection of Cort_f_ and T_f_ concentrations from a single sample (Koren *et al.*, 2012). However, steroid hormones, especially Cort_f_, remained undetected in a large proportion of samples (Koren *et al.,* 2012), which reduced the number of individuals available for statistical analysis. The reason for this could be that concentrations of Cort_f_ and T_f_ in samples were under the limits of quantifications (LOQs) of the method used. Therefore, a modification to the LC‑MS/MS method that improves hormone detectability is needed to allow the simultaneous analysis of Cort_f_ and T_f,_ from a large proportion of available samples.

References:

Bortolotti, G. R., Marchant, T. A., Blas, J. & German, T. (2008). Corticosterone in feathers is a long-term, integrated measure of avian stress physiology. *Functional Ecology*, 22, pp.494-500. doi: 10.1111/j.1365-2435.2008.01387.x

Jenni-Eiermann, S., Helfenstein, F., Vallat, A., Glauser, G. & Jenni, L. (2015). Corticosterone: effects on feather quality and deposition into feathers. *Methods in Ecology and Evolution*, 6(2), pp.237-246. doi: 10.1111/2041-210X.12314

Koren, L., Nakagawa, S., Burke, T., Soma, K., Wynne-Edwards, K. & Geffen, E. (2012). Non-breeding feather concentrations of testosterone, corticosterone and cortisol are associated with subsequent survival in wild house sparrows. *Proceedings of the Royal Society B: Biological Sciences*, 279(1733), pp.1560-1566. doi: 10.1098/rspb.2011.2062

**Appendix S3:** Field study

In 2014, a field study was carried out at three isolated farms in South Bohemia (in Lužnice (49°3'25.288"N, 14°46'10.82"E), Lomnice nad Lužnicí (49°4'7.762"N, 14°42'36.521"E) and Třeboň (48°59'05.9"N 14°46'49.5"E)), each inhabited by barn swallow colonies of roughly equal size (~ 30 breeding pairs). Adult birds were systematically captured using mist nets several times over the breeding season, with all individuals resident in the study area captured each year. Swallows were individually marked by aluminium rings with a unique number (Bird Ringing Centre, National Museum, Prague). Birds were sexed by the standard procedure (brood patch in females, cloacal protuberance in males; Svensson, 2006). Intense research and ringing of nestlings has been performed on the study plots since 2009 (e.g. Petrželková *et al.,* 2015; Safran *et al*., 2016; Kreisinger *et al.,* 2017). We used several criteria to determine the individual ages of the birds. Either the age was exactly known (in 22 birds ringed as nestlings), or it was assumed that birds newly appearing in our study area were in their second year of life (see Costanzo *et al.,* 2017). This approach is reliable in this species, which exhibits extremely high breeding philopatry (Saino, Bolzern, & Møller 1997).

References:

Costanzo, A., Ambrosini, R., Caprioli, M., Gatti, E., Parolini, M., Canova, L., … Saino, N. (2017). Lifetime reproductive success, selection on lifespan, and multiple sexual ornaments in male European barn swallows. *Evolution*, 71, 2457-2468. doi:10.1111/evo.13312

Kreisinger, J., Kropáčková, L., Petrželková, A., Adámková, M., Tomášek, O., & Martin, J. et al. (2017). Temporal Stability and the Effect of Transgenerational Transfer on Fecal Microbiota Structure in a Long Distance Migratory Bird. *Frontiers In Microbiology,* 8. doi: 10.3389/fmicb.2017.00050

Petrželková, A., Michálková, R., Albrechtová, J., Cepák, J., Honza, M., Kreisinger, J., … Albrecht, T. (2015). Brood parasitism and quasi-parasitism in the European barn swallow Hirundo rustica rustica. *Behavioral Ecology and Sociobiology*, 69, 1405-1414. doi: 10.1007/s00265-015-1953-6

Safran, R., Scordato, E., Wilkins, M., Hubbard, J., Jenkins, B., Albrecht, T., … Kane, N. (2016). Genome-wide differentiation in closely related populations: the roles of selection and geographic isolation. *Molecular Ecology*, 25(16), 3865-3883. doi: 10.1111/mec.13740

Saino, N., Bolzern, A. & Møller, A. (1997). Immunocompetence, ornamentation, and viability of male barn swallows (*Hirundo rustica*). *Proceedings of the National Academy of Sciences*, 94, 549-552. doi: 10.1073/pnas.94.2.549

Svensson, L. (2006). Identification Guide to European Passerines. Stockholm.

**Appendix S4:** Properties of LC-MS/MS analyses

The mobile phase consisted of 0.1% formic acid in water (solvent A) and 0.1% formic acid in methanol (solvent B). The mobile phase gradient was as follows: 0–2.5 min 30% B, 2.5–2.8 min from 30% B to 70% B, 70% B held to 10 min, 10–11 min from 70% B to 90% B, 90% B held to 36 min, 36–37 min from 90% B to 30% B with a subsequent equilibration step of 30% B to 50 min. The flow rate was 0.2 mL/min; 10 μL of sample was injected. The parameters of the ion source were set as follows: gas temperature – 340 °C, gas flow – 8 L/min, nebuliser – 35 psi, capillary voltage – 2000 V. Ionization was performed in positive ESI mode. The value of m/z of the precursor and product ions used in the MRM regime for quantitative and qualitative analyses can be found in Table S1 together with the fragmentor voltage and collision energies.

**Appendix S5:** Effect of derivatisation on hormone quantification

The method we introduce here is based on the precolumn chemical derivatization of hormones, these extracted from feather samples into a specific derivative using the chemical reaction of hormones with O‑(3‑trimethylammoniumpropyl) hydroxylamine bromide (QAO Reagent) (Bílková, Adámková, Albrecht, & Šimek 2019). The derivatization procedure used in our study was primarily developed for the determination of hormones in body fluids (Star-Weinstock, Williamson, Dey, Pillai, & Purkayastha 2012) and had not previously been used for the determination of hormones in feathers.

The derivatisation of hormones extracted from feather allows the sensitivity of LC-MS/MS analyses to be enhanced (Frei, 2013). Lowering LOQs also lowers the amount of sample required for the analysis. In a previously published study, more than 20 mg of feathers were used for the simultaneous analysis of T_f_ and Cort_f_ by means of LC‑MS/MS (range 21–84 mg; Koren *et al.*, 2012). Our method allows the required amount of feathers to be reduced to 4 mg and also the simultaneous analysis of T_f_ and Cort_f_ in all available samples. Such a reduction might be crucial when focusing on small-bodied passerines with a limited amount of feathers available per individual.

A typical barn swallow tail feather (with calamus removed) weighs 5.3±0.47 mg (unpublished data; M. Adámková), which means that in order to reach the 21 mg limit, one would need to utilise approximately 4 tail feathers. Such a massive removal of feathers may, however, seriously affect the targeted individual due to reduced attractiveness, flight ability, thermoregulation, or survival (McDonald & Griffith, 2011).

Although the LC‑MS/MS analysis of underivatized hormones is simpler in terms of sample preparation, there are several important reasons why one should use chemical derivatization for hormone analysis in feathers. The incomparably better identification of hormones in chromatograms of feather matrices is one obvious reason. The chemical derivatization used in our study not only allows enhancement of the MS/MS signal but also improves the specificity of detection due to the display of two pairs of characteristic peaks of isomers of T and Cort derivatives in the chromatogram of a feather sample. Therefore, our method eliminates the effect of organic contaminants in mobile phases responsible for a false positive signal and thus the high interfering responses that influence the determination of hormones in the case of methods without pre-column derivatization. The reason for this is the different retention behaviour of derivatized hormones and their specific MS/MS detection.

References:

Bílková, Z., Adámková, M., Albrecht, T. & Šimek, Z. (2019). Determination of testosterone and corticosterone in feathers using liquid chromatography-mass spektrometry. *Journal of Chromatography A*. doi: 10.1016/j.chroma.2018.12.069

Frei R. W. (Ed). (2013). *Chemical Derivatization in Analytical Chemistry*. Springer Verlag.

Koren, L., Nakagawa, S., Burke, T., Soma, K., Wynne-Edwards, K. & Geffen, E. (2012). Non-breeding feather concentrations of testosterone, corticosterone and cortisol are associated with subsequent survival in wild house sparrows. *Proceedings of the Royal Society B: Biological Sciences*, 279(1733), pp.1560-1566. doi: 10.1098/rspb.2011.2062

McDonald, P. & Griffith, S. (2011). To pluck or not to pluck: the hidden ethical and scientific costs of relying on feathers as a primary source of DNA. *Journal of Avian Biology*, 42, pp.197-203. doi: 10.1111/j.1600-048X.2011.05365.x

Star-Weinstock, M., Williamson, B., Dey, S., Pillai, S. & Purkayastha, S. (2012). LC-ESI-MS/MS Analysis of Testosterone at Sub-Picogram Levels Using a Novel Derivatization Reagent. *Analytical Chemistry*, 84, pp. 9310-9317. doi: 10.1021/ac302036r

**Table S1.** Parameters of MS/MS analyses. Precursor [M+H]^+^ and product ions, collision energies and fragmentor voltage used for multiple reaction monitoring (MRM).

| Analyte | Transition monitored | Collision energy [eV] | Fragmentor voltage [V] |
| --- | --- | --- | --- |
| Testosterone | 403.5→164.2 | 50 | 150 |
|  | 403.5→152.2 | 50 | 150 |
| Testosterone-d3 | 406.5→164.2 | 50 | 150 |
|  | 406.5→152.2 | 50 | 150 |
| Corticosterone | 288.4→369.3 | 15 | 120 |
|  | 288.4→258.9 | 20 | 120 |
| Corticosterone-d5 | 292.4→375.3 | 15 | 120 |
|  | 292.4→262.8 | 20 | 120 |

**Table S2.** Validation parameters of analyses of testosterone and corticosterone. t_R_ express the retention time of the second chromatographic peak from the pair of peaks of steric isomers.

|  | regression equation | R^2^ | [pg/injection] | | t_R_ [min] |
| --- | --- | --- | --- | --- | --- |
|  |  |  | LOD | LOQ |  |
| testosterone | y = 0.695E-03x -0.005939 | 0.9992 | 0.08 | 0.25 | 11.5 |
| corticosterone | y = 1.194E-03x-0.027955 | 0.9988 | 0.28 | 0.83 | 9.5 |

**Table S3.** Means of post-breeding concentrations of Cort_f_ and T_f_ analysed from flanks and pre-breeding concentrations of Cort_f_ and T_f_ analysed from tail feathers, expressed in picograms of hormones per gram of feather. Means are presented with accompanied standard errors (SE).

|  | post-breeding Cort_f_ | post-breeding T_f_ | pre-breeding Cort_f_ | pre-breeding T_f_ |
| --- | --- | --- | --- | --- |
| males | 1632 ± 939 | 1563 ± 509 | 1727 ± 760 | 3492 ± 660 |
| females | 1376 ± 467 | 1445 ± 420 | 1369 ± 739 | 1619 ± 901 |

**Table S4.** Full models of post- and pre-breeding hormone concentrations (log transformed) as dependent variables in relation with sex (female as a reference), age, *sex x age* interaction and sample mass. All dependent variables in the models containing significant interaction terms were centred. Predictors from the minimal adequate model are indicated in bold.

| Dependent variable | Predictor variable | Estimate | SE | df | F | P |
| --- | --- | --- | --- | --- | --- | --- |
| post-breeding log(Cort_f_) | (Intercept) | 8.719 | 0.848 |  |  |  |
|  | sex | 0.135 | 0.177 | 1 | 0.56 | 0.456 |
|  | **age** | **-0.054** | **0.06** | **1** | **3.22** | **0.075** |
|  | flank mass | -0.058 | 0.034 | 1 | 2.7 | 0.103 |
|  | sex:age | -0.04 | 0.084 | 1 | 0.22 | 0.636 |
| pre-breeding log(Cort_f_) | (Intercept) | 8.194 | 0.607 |  |  |  |
|  | sex | 0.268 | 0.224 | 1 | 7.65 | 0.007 |
|  | **age** | **-0.146** | **0.076** | **1** | **12.2** | **< 0.001** |
|  | tail mass | -0.151 | 0.108 | 1 | 1.91 | 0.169 |
|  | sex:age | -0.073 | 0.106 | 1 | 0.47 | 0.496 |
| post-breeding log(T_f_) | (Intercept) | 7.21 | 0.048 |  |  |  |
|  | **sex** | **0.069** | **0.061** | **1** | **0.775** | **0.35** |
|  | **age** | **0.068** | **0.044** | **1** | **0.491** | **0.485** |
|  | flank mass | 0.016 | 0.025 | 1 | 0.389 | 0.534 |
|  | **sex:age** | **-0.177** | **0.062** | **1** | **8.156** | **0.005** |
| pre-breeding log(T_f_) | (Intercept) | 6.863 | 0.436 |  |  |  |
|  | **sex** | **0.8** | **0.16** | **1** | **136.14** | **< 0.001** |
|  | age | -0.057 | 0.055 | 1 | 0.609 | 0.437 |
|  | tail mass | 0.093 | 0.078 | 1 | 1.39 | 0.24 |
|  | sex:age | 0.053 | 0.076 | 1 | 0.48 | 0.49 |

**Table S5.** Full model of feather growth rate as dependent variable in relation with sex (female as a reference), age, *sex x age* interaction, post- and pre-breeding hormone concentrations (log transformed) and their interactions with sex. Predictors from the minimal adequate model are indicated in bold.

| Dependent variable | Predictor variable | Estimate | SE | df | F | P |
| --- | --- | --- | --- | --- | --- | --- |
| feather growth rate | (Intercept) | 3.276 | 0.569 |  |  |  |
|  | **sex** | **-0.178** | **1.525** | **1** | **13.09** | **<0.001** |
|  | age | -0.025 | 0.036 | 1 | 0.28 | 0.598 |
|  | **pre-breeding log(Cort_f_)** | **-0.098** | **0.068** | **1** | **9.65** | **0.002** |
|  | pre-breeding log(T_f_) | -0.002 | 0.074 | 1 | 0.18 | 0.669 |
|  | sex:age | -0.024 | 0.051 | 1 | 0.11 | 0.746 |
|  | sex:pre-breeding log(Cort_f_) | -0.057 | 0.093 | 1 | 0.42 | 0.519 |
|  | sex:pre-breeding log(T_f_) | 0.06 | 0.16 | 1 | 0.14 | 0.709 |
